# Analysis of risk factors for the failure of respiratory support with high-flow nasal cannula oxygen therapy in children with acute respiratory dysfunction: A case–control study

**DOI:** 10.3389/fped.2022.979944

**Published:** 2022-08-23

**Authors:** Jie Liu, Deyuan Li, Lili Luo, Zhongqiang Liu, Xiaoqing Li, Lina Qiao

**Affiliations:** ^1^Department of Pediatric Intensive Care Unit, West China Second Universal Hospital, Sichuan University, Chengdu, China; ^2^NHC Key Laboratory of Chronobiology (Sichuan University), Ministry of Education, Chengdu, China

**Keywords:** acute respiratory insufficiency, high-flow nasal cannula oxygen therapy, risk factor, respiratory failure, pediatrics

## Abstract

**Background:**

Evidence-based clinical practice guidelines regarding high-flow nasal cannula (HFNC) use for respiratory support in critically ill children are lacking. Therefore, we aimed to determine the risk factors for early HFNC failure to reduce the failure rate and prevent adverse consequences of HFNC failure in children with acute respiratory dysfunction.

**Methods:**

Demographic and laboratory data were compared among patients, admitted to the pediatric intensive care unit between January 2017 and December 2018, who were included in a retrospective cohort study. Univariate and multivariate analyses were performed to determine risk factors for eventual entry into the predictive model for early HFNC failure and to perform an external validation study in a prospective observational cohort study from January to February 2019. Further, the association of clinical indices and trends pre- and post-treatment with HFNC treatment success or failure in these patients was dynamically observed.

**Results:**

In total, 348 pediatric patients were included, of these 282 (81.0%) were included in the retrospective cohort study; HFNC success was observed in 182 patients (64.5%), HFNC 0–24 h failure in 74 patients (26.2%), and HFNC 24–48 h failure in 26 patients (9.2%). HFNC 24 h failure was significantly associated with the pediatric risk of mortality (PRISM) III score [odds ratio, 1.391; 95% confidence interval (CI): 1.249–1.550], arterial partial pressure of carbon dioxide-to-arterial partial pressure of oxygen (PaCO_2_/PaO_2_) ratio (odds ratio, 38.397; 95% CI: 6.410–230.013), and respiratory rate-oxygenation (ROX) index (odds ratio, 0.751; 95% CI: 0.616–0.915). The discriminating cutoff point for the new scoring system based on the three risk factors for HFNC 24 h failure was ≥ 2.0 points, with an area under the receiver operating characteristic curve of 0.794 (95% CI, 0.729–0.859, *P* < 0.001), sensitivity of 68%, and specificity of 79%; similar values were noted on applying the model to the prospective observational cohort comprising 66 patients (AUC = 0.717, 95% CI, 0.675–0.758, sensitivity 83%, specificity 44%, *P* = 0.009). In this prospective cohort, 11 patients with HFNC failure had an upward trend in PaCO_2_/PaO_2_ ratio and downward trends in respiratory failure index (P/F ratio) and ROX index; however, opposite directions of change were observed in 55 patients with HFNC success. Furthermore, the fractional changes (FCs) in PaCO_2_/PaO_2_ ratio, P/F ratio, percutaneous oxygen saturation-to-fraction of inspired oxygen (S/F) ratio, and ROX index at 2 h post-HFNC therapy onset were statistically significant between the two groups (all, *P* < 0.05).

**Conclusion:**

In the pediatric patients with acute respiratory insufficiency, pre-treatment PRISM III score, PaCO_2_/PaO_2_ ratio, and ROX index were risk factors for HFNC 24 h failure, and the direction and magnitude of changes in the PaCO_2_/PaO_2_ ratio, P/F ratio, and ROX index before and 2 h after HFNC treatment were warning indicators for HFNC 24 h failure. Further close monitoring should be considered for patients with these conditions.

## Introduction

Acute respiratory insufficiency is one of the leading causes of admissions in pediatric intensive care units (PICUs) (1) requiring oxygen therapy and ventilatory support. The administration of oxygen therapy using a high-flow nasal cannula (HFNC) is a non-invasive respiratory support intervention that is easily adjustable, is well-tolerated, has a low risk of injury to the nasal mucosa and septum, and avoids the complications of invasive ventilation procedures. Many observational studies suggest that HFNC therapy is an effective modality for the early treatment of adult patients with respiratory failure associated with diverse underlying diseases, and the administration of HFNC is associated with a reduction in the rate of invasive mechanical intubation (2, 3). Furthermore, HFNC has been widely used in PICUs and has recently been considered an essential intensive care equipment (4). Several hospitals and healthcare facilities have been reported to utilize HFNC, and its use rate has increased to 77% in 2018 from 28.1% in 2017 in the United States (5) and 63% in Australia and New Zealand (2). Recently, HFNC has been increasingly utilized for the respiratory management of bronchiolitis, acute asthma, and other respiratory distress (4, 6). Moreover, HFNC reportedly helps avoid intubation in hypoxemic patients suffering from coronavirus disease 2019 (COVID-19) (7, 8). However, no guidelines regarding the indications and contraindications for HFNC therapy in pediatric patients currently exist, and there is a consensus that the overuse and misuse of HFNC have contributed to the serious adverse event of HFNC failure (9). Some large-sample studies involving adult patients with respiratory failure revealed that HFNC failure was associated with increased mortality rates, compared with bilevel positive airway pressure (BiPAP) and invasive mechanical ventilation (IMV) alone, and that the extended use of HFNC before administering intubation may be deleterious (10, 11). In the pediatric field, a retrospective study by Taha et al. observed that very-low birthweight infants who received HFNC therapy experienced respiratory support failure with an increased risk of death, bronchopulmonary dysplasia (BPD), respiratory system disorders, and extended length of hospital stay (12). Moreover, recent data (5, 6) have demonstrated a trend toward increased mortality in patients who experienced HFNC failure and required intubation, and one possible explanation is that practitioners are not yet fully informed regarding the best candidate for HFNC use. This underscores the necessity of clear guidelines and evidence-based information regarding the indications for HFNC therapy to prevent misapplication, which may potentially harm patients, particularly critically ill pediatric patients.

Recently, an increasing number of studies have revealed that intubation following early HFNC failure (before 48 h) is associated with lower mortality rate in adult patients with acute respiratory failure, and in patients who respond to HFNC treatment generally, an improvement is observed within the first 1–2 h, with the highest risk of treatment failure being observed within the first 24 h (4, 11, 13). However, data regarding the risk factors for HFNC failure in critically ill children are scarce, and evidence-based clinical practice guidelines for HFNC use for respiratory support in critically ill children are currently lacking. Previously, we have reported that the pediatric risk of mortality (PRISM) III score and arterial partial pressure of carbon dioxide-to-arterial partial pressure of oxygen (PaCO_2_/PaO_2_) ratio were significantly associated with HFNC 48 h failure (14). Thus, this study aimed to identify the risk factors for HFNC 24 h failure to further reduce the serious outcomes of early HFNC failure in critically ill pediatric patients at high risk of respiratory deterioration.

## Materials and methods

### Study design and population

We retrospectively reviewed the clinical records of consecutive children with acute respiratory insufficiency who received respiratory support synchronously directly after admission to the PICU of the West China Second University Hospital of Sichuan University from January 2017 to December 2018. Clinical indicators before and after HFNC treatment were dynamically observed in an independent prospective observational cohort from January to February 2019. The criteria for the diagnosis of acute respiratory insufficiency were as follows: signs of respiratory distress including tachypnea (increased respiratory rate in infants < 2 months: ≥ 60 breaths/min; 2–12 months: ≥ 50 breaths/min; 1–5 years: ≥ 40 breaths/min; and ≥ 5 years: ≥ 30 breaths/min), oral cyanosis, retractions or auxiliary respiratory muscles participating in the respiratory movement, positive findings for the three concave signs (suprasternal fossa, supraclavicular fossa, and intercostal space) during inspiration, cough, loss of consciousness or restlessness, fatigue, and tachycardia in a patient. Blood gas analysis without oxygen inhalation included an arterial partial pressure of oxygen (PaO_2_) < 8.0 kPa (60 mmHg) and/or an arterial partial pressure of carbon dioxide (PaCO_2_) > 6.67 kPa (50 mmHg), or a respiratory failure index (P/F ratio) ≤ 300 with oxygen inhalation; patients who maintained percutaneous oxygen saturation (SpO_2_) between 88 and 92% using a nasal catheter or mask were also included (15–17). As other kinds of non-invasive ventilation, such as continuous positive airway pressure (CPAP) or BiPAP, are not employed on a routine basis as the second line of ventilatory support in the event of HFNC failure at our medical center, HFNC failure was defined if patients satisfied the following criteria (18): the presence of loss of consciousness, dysphoria, dyspnea, blood oxygen saturation < 90%, or carbon dioxide retention that failed to improve within 2 h of HFNC therapy (administered at an oxygen concentration ≥ 60%, oxygen flow ≥ 2 L/kg/min, and a maximum of ≤ 60 L/min), which was required to upgrade the respiratory support mode to IMV, and the final decisions regarding HFNC discontinuation and IMV initiation were made collaboratively by the respiratory therapist and the physicians in charge. Patients were excluded if they had cardiac or respiratory arrest requiring emergency endotracheal intubation or mechanical ventilation, weak spontaneous respiration or an P/F ratio < 100 mmHg, upper airway obstruction, facial trauma, deformity, or poor upper airway protection; children with HFNC intolerance, difficulty in removing large amounts of sputum or risk of aspiration, who previously received respiratory support outside the hospital, who were voluntarily discharged within 24 h after admission or received upgraded respiratory support after 48 h of HFNC treatment, or had incomplete data required for statistical analyses were also excluded.

All enrolled patients received resuscitation measures and basic treatment to actively control the primary disease, and HFNC therapy was administered within 15 min after admission. The inhaled gas temperature was 37°C, and oxygen was administered for 24 h without interruption. The parameters were adjusted according to the blood gas analysis results; the PaO_2_ was maintained at 60–80 mmHg (8.0–10.67 kPa), PaCO_2_ was maintained at 40–50 mmHg (5.33–6.67 kPa), and SpO_2_ was maintained > 90%. When the target SpO_2_ was maintained and the condition of the patients improved, inspiratory oxygen fraction (FiO_2_) was gradually reduced to 21–25%, and HFNC therapy was withdrawn if the patient was stable for 4–6 h. According to their response to HFNC treatment and the time of respiratory support upgrade, the retrospective cohort with 282 patients was divided into three groups as follows: the success group (*n* = 182), 0–24 h failure group (*n* = 74), and 24–48 h failure group (*n* = 26), and the association between baseline characteristics with HFNC 24 h failure was investigated among the groups. To further validate the predictive value of the risk factors for HFNC 24 h failure, we used another prospective observational cohort comprising 66 patients (55 successful vs. 11 failed) and dynamically observed the clinical indices and trends before and after treatment to identify baseline warning signs of 24 h treatment failure within 2 h of starting the initial HFNC therapy.

### Data collection

The following clinical and laboratory data collected from the medical charts of the patients enrolled in this study were reviewed using a standardized form: (i) demographic and clinical data: age (in years) at disease onset, sex, weight, body mass index (BMI), and Glasgow coma scale (GCS) score on the day of admission; (ii) laboratory data: blood samples were collected from 282 patients upon admission to the PICU before HFNC treatment for laboratory tests, including arterial blood gas, complete blood count, C-reactive protein (CRP), procalcitonin (PCT), serum electrolytes, random blood glucose, liver function, kidney function, and coagulation function tests. We calculated the PRISM III score, PaCO_2_/PaO_2_ ratio, P/F ratio, percutaneous oxygen saturation-to-fraction of inspired oxygen (S/F) ratio, and respiratory rate-oxygenation (ROX) index based on the aforementioned indicators. Additionally, another 66 blood samples were collected for arterial blood gas analysis before and at 2, 6, and 12 h following initiation of HFNC treatment. The fractional change (FC) of variables that indicated a significant difference between the groups was also compared. FC was defined as FC = (Y – X)/X, where X represents data before HFNC treatment, and Y represents data 2 h after HFNC treatment.

### Statistical analyses

Normality of distribution was verified using the Shapiro–Wilk and homogeneity tests. Data with a normal distribution are expressed as mean ± standard deviation, and the two-independent sample *t*-test or one-way analysis of variance was used to compare data between groups. Measurement data without a normal distribution are expressed as median (four-digit interval) [P_50_ (P_25_, P_75_)], and these data were compared between groups using the Mann–Whitney U or Kruskal–Wallis *H*-test. Enumeration data were expressed as percentages (%). The Fisher’s exact test, Chi-square test, or Pearson’s Chi-square test was used to perform intergroup comparisons, and the Bonferroni correction was applied for multiple comparisons. Variance inflation factors were used to check for collinearity, significant indices were analyzed using multivariate logistic regression to determine risk factors, and the optimum threshold for the significant parameter was constructed using receiver operating characteristic (ROC) curves. Numerical variables that indicated significance in the multivariate logistic regression analysis were transformed into dichotomous variables, and the score point of each predictor was determined using the value of the logistic coefficient. The area under the curve (AUC) was calculated to evaluate the capacity of the model, and the *P*-values were two-tailed, with *P* < 0.05 considered significant. Statistical analyses were performed using SPSS, version 26.0 (IBM Corp., Armonk, NY, United States).

## Results

### Baseline characteristics

**FIGURE 1:**
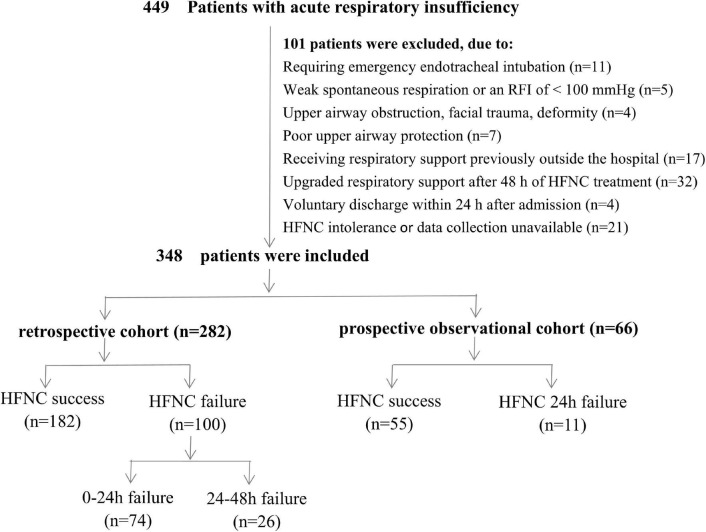
Flow chart of the study. HFNC, high-flow nasal cannula.

Over the period of observation, 449 children with acute respiratory insufficiency were admitted to the PICU of West China Second Universal Hospital. In total, 101 children were excluded from this study, including 27 children who had cardiac or respiratory arrest requiring emergency endotracheal intubation or mechanical ventilation, weak spontaneous respiration or a P/F ratio < 100 mmHg, upper airway obstruction, facial trauma, deformity, or poor upper airway protection; 17 children who received respiratory support previously outside the hospital; 36 children who were voluntarily discharged within 24 h after admission or received upgraded respiratory support after 48 h of HFNC treatment; and 21 with HFNC intolerance or incomplete clinical or laboratory data. Ultimately, 348 children were included in this study (282 patients in the retrospective cohort and 66 in the prospective observational cohort), and HFNC failure was noted in 111 (31.9%) patients (Figure 1). The initial parameters were FiO_2_ range of 0.3–1.0 and flow range of 2–3 L/kg/min for patients with HFNC success, and FiO_2_ range of 0.4–1.0 and flow range of 2–3 L/kg/min for patients with HFNC failure. In addition, the primary diseases of patients in the included studies were severe pneumonia (*n* = 141, 40.5%), sepsis (*n* = 59, 17.0%), shock (*n* = 35, 10.1%), multiple organ dysfunction syndrome (*n* = 33, 9.5%), intracranial hypertension syndrome (38, 10.9%), BPD with pulmonary infection (*n* = 30, 8.6%), ARDS (*n* = 5, 1.4%), chemotherapy-induced myelosuppression with infection (*n* = 5, 1.4%), and pneumorrhagia (*n* = 2, 0.6%). The distribution of disease types was not significantly different between the success and the failure groups (Supplementary Table 1).

### Intragroup comparisons between high-flow nasal cannula success and failure groups to analyze the risk factors for early high-flow nasal cannula failure

#### Comparison of the baseline characteristics

Altogether, 282 patients admitted to the PICU were enrolled in the retrospective cohort. Of these, 182 (64.5%) patients had HFNC success, 74 (26.2%) had HFNC 0–24 h failure, and 26 (9.2%) had HFNC 24–48 h failure. The PRISM III score and PaCO_2_/PaO_2_ ratio were higher in the 0–24 h failure group than in the success group, while the GCS score, P/F ratio, and ROX index of the failure group were lower than those of the success group. Additionally, the S/F ratio in the 0–24 h failure group was lower than that in the remaining groups (*P* < 0.05). However, no significant differences in age, sex, weight, BMI, CRP, PCT, blood lactate, pH, PaCO_2_, and PaO_2_ were observed between the groups (*P* > 0.05 for all; Table 1).

**TABLE 1:** Baseline characteristics of patients in the high-flow nasal cannula oxygen therapy success and failure groups.

	Success (*n* = 182)	0–24 h failure (*n* = 74)	24–48 h failure (*n* = 26)
Age [year, *P*_50_ (*P*_25_, *P*_75_)]	0.50 (0.17, 1.50)	0.96 (0.17, 5.00)	0.34 (0.08, 0.89)
Male [*n*(%)]	107 (58.8)	36 (48.6)	10 (38.5)
Weight [kg, *P*_50_ (*P*_25_, *P*_75_)]	6.80 (4.78, 10.70)	7.80 (4.38, 15.85)	5.30 (4.03, 7.90)
BMI [kg/m^2^, mean ± SD]	14.74 ± 2.13	14.37 ± 2.51	13.80 ± 3.22
GCS score [point, *P*_50_ (*P*_25_, *P*_75_)]	13.00 (13.00, 14.00)	12.00 (10.00, 14.00)*	13.00 (12.00, 14.00)
PRISM III score [point, *P*_50_ (*P*_25_, *P*_75_)]	1.00 (0.00, 3.00)	4.50 (1.75, 9.00)*	3.00 (1.00, 5.25)^†^
CRP [mg/L, ref. 0–10 mg/L], *P*_50_ (*P*_25_, *P*_75_)	5.00 (2.00, 26.08)	9.00 (2.00, 46.25)	5.00 (2.00, 15.00)
PCT [ref. 0–0.05, *P*_50_ (*P*_25_, *P*_75_)]	0.36 (0.15, 1.44)	0.67 (0.21, 4.99)	0.55 (0.16, 1.22)
Blood lactate [mmol/L, ref. 0.63–2.44 mmo/L], *P*_50_ (*P*_25_, *P*_75_)	1.70 (1.30, 2.51)	1.85 (1.30, 3.30)	1.80 (1.37, 3.15)
pH [ref. 7.35–7.45, *P*_50_ (*P*_25_, *P*_75_)]	7.40 (7.36, 7.43)	7.36 (7.31, 7.40)	7.39 (7.34, 7.44)
PaCO_2_ [mmHg, ref. 35–45 mmHg], *P*_50_ (*P*_25_, *P*_75_)	38.70 (33.38, 46.55)	42.95 (32.10, 61.28)	44.45 (34.13, 53.60)
PaO_2_ [mmHg, ref. 80–100 mmHg], *P*_50_ (*P*_25_, *P*_75_)	83.35 (67.48, 100.55)	76.00 (62.14, 100.50)	73.70 (57.68, 99.13)
PaCO_2_/PaO_2_ ratio [*P*_50_ (*P*_25_, *P*_75_)]	0.48 (0.36, 0.62)	0.61 (0.35, 0.75)*	0.62 (0.39, 0.85)
P/F ratio [mmHg, ref. 400–500 mmHg], mean ± SD	223.18 ± 71.59	161.36 ± 78.67*	193.76 ± 61.81
S/F ratio [*P*_50_ (*P*_25_, *P*_75_)]	237.50 (180.00, 300.00)	156.67 (123.75, 207.31)*^‡^	204.55 (173.33, 384.79)
ROX [mean ± SD]	5.14 ± 2.60	4.10 ± 2.57*	4.86 ± 2.46

*P < 0.05 in success group vs. 0–24 h failure group. ^†^P < 0.05 in success group vs. 24–48 h failure group. ^‡^P < 0.05 in 0–24 h failure group vs. 24–48 h failure group.

BMI, body mass index; GCS, Glasgow Coma Scale; PRISM, pediatric risk of mortality; CRP, C-reactive protein; PCT, procalcitonin; pH, pondus hydrogenii; PaCO_2_, arterial partial pressure of carbon dioxide; PaO_2_, arterial partial pressure of oxygen; PaCO_2_/PaO_2_, arterial partial pressure of carbon dioxide-to-arterial partial pressure of oxygen; P/F ratio, arterial partial oxygen pressure-to-fraction of inspired oxygen ratio; S/F ratio, percutaneous oxygen saturation-to-fraction of inspired oxygen ratio; ROX, ratio of percutaneous oxygen saturation and fraction of inspired oxygen to respiratory rate.

#### Results of the multi-factor logistic analysis

To determine the relative effect of each risk factor for early HFNC failure in PICU patients, we performed a logistic regression analysis, which revealed that HFNC 24 h failure was significantly associated with six baseline variables (GCS score, PRISM III score, PaCO_2_/PaO_2_ ratio, P/F ratio, and ROX index, and S/F ratio). The multivariable analysis included the PRISM III score instead of GCS score because the former contained the latter, and the ROX index, calculated as the S/F ratio divided by the respiratory rate, was included in the multivariable analysis instead of the separate indicator. All variables were tested for collinearity; however, no collinearity was present. The PRISM III score [odds ratio, 1.391; 95% confidence interval (CI): 1.249–1.550], PaCO_2_/PaO_2_ ratio (odds ratio, 38.397; 95% CI: 6.410–230.013), and ROX index (odds ratio, 0.751; 95% CI: 0.616–0.915) were significantly associated with HFNC 24 h failure after correction for age and sex. The PRISM III score and PaCO_2_/PaO_2_ ratio were also related to HFNC 48 h failure (odds ratio: 1.247, 25.401, respectively) (Table 2).

**TABLE 2:** Relationship between risk factors and high-flow nasal cannula oxygen therapy failure, compared to success group.

	Univariate				Multivariate				Adjust^#^				VIF
	0–24 h Failure		24–48 h Failure		0–24 h Failure		24–48 h Failure		0–24 h Failure		24–48 h Failure		
	Odds ratio (95%CI)	*P*-value	Odds ratio (95%CI)	*P*-value	Odds ratio (95%CI)	*P*-value	Odds ratio (95%CI)	*P*-value	Odds ratio (95%CI)	*P*-value	Odds ratio (95%CI)	*P*-value	
PRISM III score	1.371 (1.249–1.505)	<0.001	1.218 (1.075–1.379)	0.002	1.396 (1.256–1.552)	<0.001	1.249 (1.091–1.429)	0.001	1.391 (1.249–1.550)	<0.001	1.247 (1.089–1.427)	0.001	1.093
PaCO_2_/PaO_2_ ratio	9.359 (2.844–30.793)	<0.001	8.338 (1.951–35.643)	0.004	14.810 (2.967–73.922)	0.001	15.064 (2.529–89.732)	0.003	38.397 (6.410–230.013)	<0.001	25.401 (3.492–184.736)	0.001	1.183
P/F ratio	0.987 (0.982–0.992)	<0.001	0.994 (0.989–1.000)	0.065	0.995 (0.989–1.001)	0.097	0.998 (0.991–1.005)	0.628	0.998 (0.992–1.004)	0.591	1.000 (0.993–1.007)	0.988	1.615
ROX	0.829 (0.728–0.945)	0.005	0.962 (0.822–1.128)	0.636	0.858 (0.723–1.019)	0.081	0.953 (0.785–1.156)	0.624	0.751 (0.616–0.915)	0.005	0.906 (0.725–1.132)	0.385	1.411

^#^ Indicates a significant relationship after correction for age and sex. VIF, variance inflation factors; PRISM, pediatric risk of mortality; PaCO_2_/PaO_2_, arterial partial pressure of carbon dioxide-to-arterial partial pressure of oxygen; P/F ratio, arterial partial oxygen pressure-to-fraction of inspired oxygen ratio; ROX, ratio of percutaneous oxygen saturation and fraction of inspired oxygen to respiratory rate.

#### Predictive model for high-flow nasal cannula 24 h failure

**FIGURE 2:**
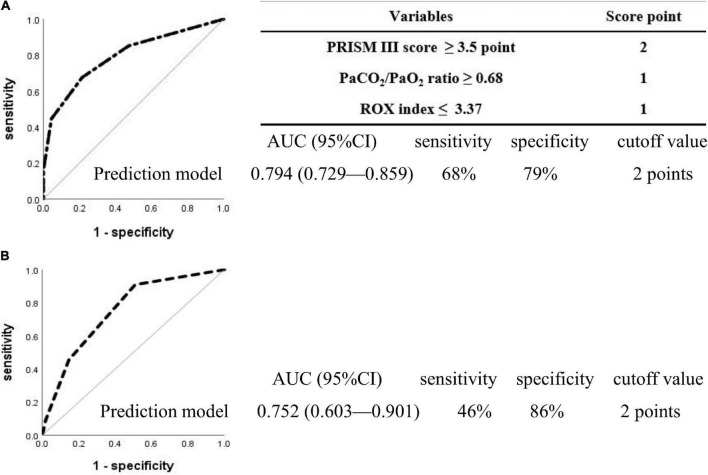
Receiver operating characteristic curves for the prediction model in the patients with 24 h high-flow nasal cannula failure. Two hundred and eighty-two patients were included in the retrospective cohort **(A)**, 66 in the prospective observational cohort **(B)**, and no overlap was observed between the two cohorts. The discriminated cutoff point for the new scoring system to predict HFNC 24 h failure was ≥ 2 points, with an AUC of 0.794 (95% CI, 0.729–0.859, *P* < 0.001), sensitivity of 68%, and specificity of 79% in the retrospective cohort **(A)**, and with an AUC of 0.752 (95% CI, 0.603–0.901, *P* = 0.009), sensitivity of 46%, and specificity of 86% in prospective observational cohort **(B)**. PRISM, pediatric risk of mortality; PaCO_2_/PaO_2_, arterial partial pressure of carbon dioxide-to-arterial partial pressure of oxygen; ROX, ratio of percutaneous oxygen saturation and fraction of inspired oxygen to respiratory rate; AUC, area under the curve; CI, confidence interval.

The cutoff values of the parameters were determined using ROC curves and are presented in the Supplementary File. The PRISM III score cutoff value of 3.5 points yielded a sensitivity of 60% and a specificity of 82% [AUC = 0.769 (95% CI, 0.704–0.834, *P* < 0.001]; the PaCO_2_/PaO_2_ ratio cutoff value of 0.68 yielded 43% sensitivity and 87% specificity [AUC = 0.604 (95% CI, 0.517–0.691, *P* = 0.009)]; and the ROX index cutoff value of 3.37 yielded 51% sensitivity and 76% specificity [AUC = 0.650 (95% CI, 0.575–0.725, *P* < 0.001)]. Stratified analysis based on the three parameters with their respective cutoff values was performed, and the scores for each variable were obtained (a score of 2 points for the PRISM III score ≥ 3.5 points, a score of 1 point for the PaCO_2_/PaO_2_ ratio ≥ 0.68, and 1 point for the ROX index ≤ 3.37, totaling to a maximum of 4 points). The discriminating cutoff point for the new scoring system to predict HFNC 24 h failure was ≥ 2.0 points, with an AUC of 0.794 (95% CI, 0.729–0.859, *P* < 0.001), a sensitivity of 68%, and a specificity of 79% (Figure 2A), and with an AUC of 0.752 (95% CI, 0.603–0.901, *P* = 0.009), a sensitivity of 46%, and a specificity of 86% in the prospective cohort comprising 66 patients (Figure 2B).

#### Baseline warning signs of high-flow nasal cannula 24 h failure within 2 h after the initial high-flow nasal cannula therapy onset

**FIGURE 3:**
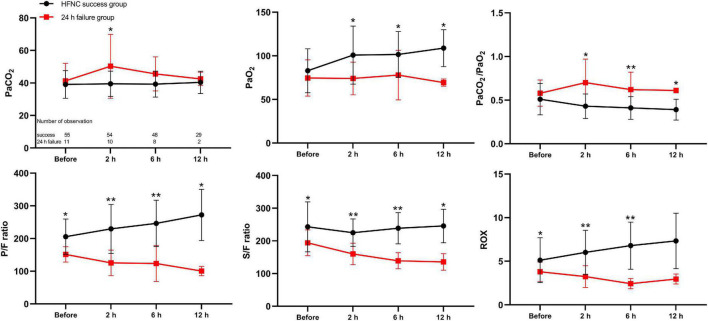
Time-course changes of arterial blood gas analysis before and 2, 6, and 12 h after high-flow nasal cannula treatment onset as mean (SD) in the prospective cohort. CI, confidence interval; PaCO_2_, arterial partial pressure of carbon dioxide; PaO_2_, arterial partial pressure of oxygen; PaCO_2_/PaO_2_, arterial partial pressure of carbon dioxide-to-arterial partial pressure of oxygen; P/F ratio, arterial partial oxygen pressure-to-fraction of inspired oxygen ratio; S/F ratio, percutaneous oxygen saturation-to-fraction of inspired oxygen ratio; ROX, ratio of percutaneous oxygen saturation and fraction of inspired oxygen to respiratory rate. *P < 0.05; **P < 0.001.

**FIGURE 4:**
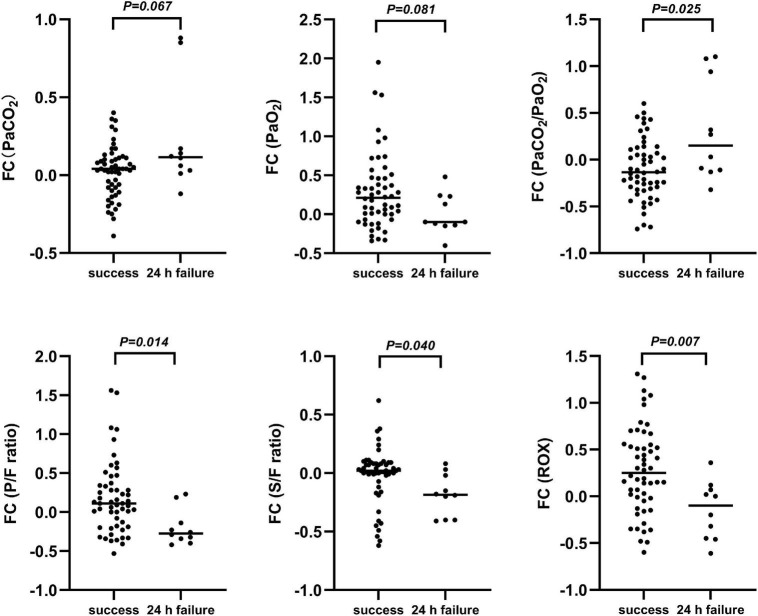
Fractional changes in blood gas indexes prior to treatment and up to 2 h post-treatment. FC, Fractional change = (data 2 h after HFNC treatment)-(data before HFNC treatment)/data before HFNC treatment; PaCO_2_, arterial partial pressure of carbon dioxide; PaO_2_, arterial partial pressure of oxygen; PaCO_2_/PaO_2_, arterial partial pressure of carbon dioxide-to-arterial partial pressure of oxygen; P/F ratio, arterial partial oxygen pressure-to-fraction of inspired oxygen ratio; S/F ratio, percutaneous oxygen saturation-to-fraction of inspired oxygen ratio; ROX, ratio of percutaneous oxygen saturation and fraction of inspired oxygen to respiratory rate.

Of the patients, 66 admitted to the PICU were enrolled in the prospective observational cohort study, among whom 55 had successful therapy and 11 had failed therapy. Among the patients who required escalation of respiratory support, seven (63.6%) were upgraded within 12 h and four (36.3%) for the period of 12–24 h after HFNC therapy onset. The mean time of upgrading respiratory support was 11.6 h (range, 1–23 h). The baseline characteristics and results of the arterial blood gas analysis before and 2, 6, and 12 h after HFNC treatment onset by study group are presented in Supplementary Table 2. Changes in oxygenation status were observed and compared with baseline data, and patients with HFNC failure had an upward trend in the PaCO_2_/PaO_2_ ratio and downward trends in the P/F ratio and ROX index (Figure 3). Opposite directions of change were observed in patients with HFNC success, and the FCs in PaCO_2_/PaO_2_ ratio, P/F ratio, S/F ratio, and ROX index were significant at 2 h after HFNC therapy onset between patients with and without HFNC failure (*P* < 0.05 for all) (Figure 4).

## Discussion

Our findings revealed that the PRISM III score, PaCO_2_/PaO_2_ ratio, and ROX index were significantly associated with an increased risk of HFNC 24 h failure. In addition, we observed that the direction and magnitude of changes in the PaO_2_/FiO_2_ ratio, P/F ratio, and ROX index before and 2 h after HFNC treatment are warning indicators of HFNC 24 h failure. Thus, we recommend that patient selection should be carefully considered clinically before HFNC administration and that patients should be monitored critically to identify and treat HFNC failure.

HFNC has recently emerged as an effective alternative to non-invasive respiratory support in the treatment of patients with acute respiratory failure; however, early prediction of HFNC failure is crucial, as most patients (74.0%) received upgraded respiratory support within 24 h of HFNC therapy initiation in the retrospcetive cohort involving 100 patients with HFNC failure in our study, in accordance with previous findings (19–23). Disease severity and poor initial response to HFNC treatment have been demonstrated to be closely related to HFNC failure by multiple studies (24, 25); among them, organ dysfunction has been reported to be a risk factor for HFNC failure (26), which is consistent with our findings that a greater PRISM III score was associated with a considerably increased risk of HFNC failure. PRISM III score, a physiology-based measurement that involves 14 physiological parameters and 23 parameter ranges (27), including arterial blood gas, blood sugar, electrolytes, liver and kidney function tests, and coagulation function tests, is the most common currently available system used for mortality prediction in the PICU. A study in China has shown that PRISM III score is a robust indicator of prognosis in critically ill children (28). However, generating a PRISM III score is more time-consuming than generating other existing warning scores. For example, the pediatric early warning system (PEWS) score limits the power of the PRISM III score in predicting early HFNC failure. An in-depth study to identify a more valuable scoring system is recommended.

Despite the lack of established guidelines for the use of HFNC in hypercapnic respiratory failure, HFNC can provide a constant oxygen concentration and a low level of positive end-expiratory pressure in the airway with high-flow gas to improve oxygenation. HFNC can reduce the anatomical dead space and improve carbon dioxide wash-out (29), indicating that HFNC therapy may be beneficial for treating not only hypoxemic but also acute hypercapnic respiratory failure (30–32). By contrast, Sztrymf et al. have reported that moderate PaCO_2_ increased after HFNC treatment (33, 34), illustrating that HFNC application in hypercapnia is controversial. PaCO_2_, which is the numerator, had a negative association with HFNC success, whereas PaO_2_, which is the denominator, had a positive relationship with HFNC success. The PaCO_2_/PaO_2_ ratio is easy to use and could indicate pulmonary ventilation and diffusion function, and outperform the diagnostic accuracy of the two variables separately, as confirmed in our study. Interestingly, this study identified that PRISM III score and PaCO_2_/PaO_2_ ratio were significantly associated with HFNC 24 h failure, as well as 48 h failure, and this is consistent with our previous results (14). It is worth noting that an increasing trend of PaCO_2_/PaO_2_ ratio was observed in patients with HFNC failure alongside the decreasing pattern in patients with HFNC success after treatment, which may help pediatricians distinguish patients who will experience HFNC success from those who will experience HFNC failure. To the best of our knowledge, this is the first study to suggest that a high PaCO_2_/PaO_2_ ratio is a risk factor for HFNC failure. Nevertheless, future large-scale studies are required to verify these findings.

ROX index, defined as the ratio of SpO_2_/FiO_2_ to respiratory rate, has been reported to have a high predictive value to identify HFNC failure in critically ill patients with acute hypoxemic respiratory failure (AHRF) (35). A study published 2 years later discovered that combining pediatric ROX index (p-ROXI) and its dynamic alterations can successfully predict HFNC failure within 24 h and 48 h of therapy onset (36). However, the respiratory rate z-score, instead of respiratory rate, is not routinely used clinically and is cumbersome and decreased the value as a bedside monitoring indicator. Moreover, a recent prospective observational study has reported that elevated respiratory drive (a better predictor for HFNC failure) increases tidal volume (VT), but not respiratory rate. Therefore, the overall discriminatory ability of volume-oxygenation index (SpO_2_/FiO_2_ to VT) was superior to that of ROX index in identifying ICU patients with AHRF and HFNC failure within 12 h following initiation of HFNC treatment (37). However, measurement of VT in children is more difficult than in adults, and is yet to be performed in our centers; hence, p-ROXI and SpO_2_/FiO_2_ to VT were not applied in this study. Noteworthily, ROX index may serve as a good predictive marker for HFNC failure when it was measured within the first 24 h of HFNC therapy during the ongoing global COVID-19 outbreak (38). Here, the ROX index was higher in the success group than in the failed group before and 2, 6, and 12 h after HFNC treatment. The multivariate logistic regression analysis revealed that ROX index was considered a risk factor for HFNC 24 h failure, and we generated a new scoring system based on the three risk factors (PRISM III score, PaCO_2_/PaO_2_ ratio, and ROX index) for HFNC 24 h failure, with a comparable predictive value when applied to another cohort. Furthermore, the direction of change in ROX index demonstrated a downward trend in the failed group but an upward trend in the success group. Similar results were observed for the direction of change in P/F ratio, and all these dynamic changes revealed that oxygenation improved in patients who respond to the HFNC treatment, whereas HFNC non-responders reached a plateau in the improvement of their oxygenation index within the first 12 h of HFNC therapy onset. However, the sample size for the dynamic changes in arterial blood gas analysis was insignificant for the multivariable regression analysis; therefore, statistical power may be limited, and future large-scale studies are required to verify the association between ROX index and HFNC failure when measured prospectively.

Our data have several limitations. First, the sample size was too small to allow sub-group analyses stratified by different disease groups and to unequivocally determine the dynamic changes in the predictors of early HFNC failure. Second, this study did not evaluate dynamic changes after 2 h post-HFNC treatment due to missing data; additionally, the small sample size and wide CIs limited the statistical power. Hence, these findings should be interpreted with caution, and further multicenter studies with adequate sample sizes are warranted to assess the generalizability of these findings. Third, the PRISM III scoring system is time-consuming, thus limiting its power in predicting early HFNC failure, and some instantaneous evaluation indicators, such as the PEWS score, were not considered in the initial study design. Hence, more prospective studies are required for validation of a highly determinant vital sign that is easily measured at the bedside for predicting HFNC failure in critically ill pediatric patients.

## Conclusion

In conclusion, in pediatric patients with acute respiratory insufficiency, pre-treatment PRISM III score, PaCO_2_/PaO_2_ ratio, and ROX index were risk factors for HFNC 24 h failure. In addition, the direction and magnitude of changes in the PaCO_2_/PaO_2_ ratio, P/F ratio, and ROX index before and 2 h after HFNC treatment are warning indicators for HFNC 24 h failure. Further close monitoring should be considered for patients with these conditions. Future studies with a larger sample per disease subgroup are required to verify the association of these risk factors with HFNC failure.

## Data availability statement

The original contributions presented in this study are included in the article/Supplementary material, further inquiries can be directed to the corresponding author/s.

## Ethics statement

Written informed consent was obtained from the individual(s), and minor(s)’ legal guardian/next of kin, for the publication of any potentially identifiable images or data included in this article.

## Author contributions

JL, DL, and LQ: conceptualization. JL, LL, and ZL: formal analysis. ZL and XL: methodology. JL and DL: writing—original draft. JL and LQ: writing—review and editing. All authors approved the final manuscript to be submitted and agreed to be accountable for all aspects of the work.

## Abbreviations

AHRF: acute hypoxemic respiratory failure
ARDS: acute respiratory distress syndrome
AUC: area under the curve
BPD: bronchopulmonary dysplasia
COVID-19: coronavirus disease 2019
CRP: C-reactive protein
FC: fractional change
FiO_2_: inspired oxygen fraction
GCS: Glasgow coma scale
HFNC: high-flow nasal cannula
IMV: invasive mechanical ventilation
PCT: platelet count
PEWS: pediatric early warning system
PICU: pediatric intensive care unit
PRISM: pediatric risk of mortality
P/F ratio: respiratory failure index
ROC: receiver operating characteristic curve
ROX: respiratory rate-oxygenation
SpO_2_: percutaneous oxygen saturation
S/F ratio: the ratio of percutaneous oxygen saturation to inspiratory oxygen fraction.
