# Supplementary material for: Analysis of risk factors for the failure of respiratory support with high-flow nasal cannula oxygen therapy in children with acute respiratory dysfunction: A case–control study
Source: Front Pediatr. 2022 Aug 23;10:979944. doi: 10.3389/fped.2022.979944 (PMC9445578; doi:10.3389/fped.2022.979944)
Supplement: Supplementary file 1 [file Table_1.docx]

Supplementary Table 1

Disease distribution of patients with high-flow nasal cannula oxygen therapy success and failure groups

|  | Success (n=182) | 0–24 h failure (n=74) | 24–48 h failure (n=26) | *P-value* |
| --- | --- | --- | --- | --- |
| Severe pneumonia [n(%)] | 75 (41.2) | 19 (25.7) | 9 (34.6) | 0.063 |
| Sepsis [n(%)] | 31 (17.0) | 14 (18.9) | 4 (15.4) | 0.901 |
| Shock [n(%)] | 23 (12.6) | 7 (9.5) | 2 (7.7) | 0.621 |
| Multiple organ dysfunction syndrome [n(%)] | 15 (8.2) | 12 (16.2) | 2 (7.7) | 0.171 |
| Intracranial hypertension syndrome [n(%)] | 20 (11.0) | 8 (10.8) | 3 (11.5) | 0.995 |
| Bronchopulmonary dysplasia with pulmonary infection [n(%)] | 14 (7.7) | 7 (9.5) | 5 (19.2) | 0.225 |
| Acute respiratory distress syndrome [n(%)] | 1 (0.5) | 3 (4.1) | 1 (3.8) | 0.059 |
| Chemotherapy-induced myelosuppression with infection [n(%)] | 2 (1.1) | 3 (4.1) | 0 | 0.181 |
| Pneumorrhagia [n(%)] | 1 (0.5) | 1 (1.4) | 0 | 0.580 |
