# Supplementary material for: Analysis of risk factors for the failure of respiratory support with high-flow nasal cannula oxygen therapy in children with acute respiratory dysfunction: A case–control study
Source: Front Pediatr. 2022 Aug 23;10:979944. doi: 10.3389/fped.2022.979944 (PMC9445578; doi:10.3389/fped.2022.979944)
Supplement: Supplementary file 2 [file Table_2.docx]

Supplementary Table 2

Baseline characteristics of patients with high-flow nasal cannula oxygen therapy success and 24 h failure groups

|  | Success (n=55) | | 24 h failure (n=11) | | *P-value* |
| --- | --- | --- | --- | --- | --- |
| Demographic characteristics | | | | | |
| Age [year,  *P*_50_ (*P*_25_, *P*_75_)] | 55 | 0.42 (0.17, 2.00) | 11 | 0.92 (0.58, 4.42) | 0.286 |
| Male [n(%)] | 55 | 31 (56.4) | 11 | 5 (45.5) | 0.507 |
| Weight [kg,  *P*_50_ (*P*_25_, *P*_75_)] | 55 | 7.00 (5.20, 10.60) | 11 | 8.80 (6.00, 17.00) | 0.547 |
| BMI [kg/m^2^, mean±SD] | 55 | 14.76±2.28 | 11 | 14.81±1.06 | 0.951 |
| GCS score [point, mean±SD] | 55 | 13.09±1.47 | 11 | 13.18±0.98 | 0.845 |
| PRISM III score [point, *P*_50_ (*P*_25_, *P*_75_)] | 55 | 0.00 (0.00, 2.00) | 11 | 1.00 (0.00, 7.00) | 0.040 |
| Disease distribution | | | | | |
| Severe pneumonia [n(%)] |  | 34 (61.8) |  | 4 (36.4) | 0.220 |
| Sepsis [n(%)] |  | 7 (12.7) |  | 3 (27.3) | 0.443 |
| Shock [n(%)] |  | 2 (3.6) |  | 1 (9.1) | 0.427 |
| Multiple organ dysfunction syndrome [n(%)] |  | 3 (5.5) |  | 1 (9.1) | 0.527 |
| Intracranial hypertension syndrome [n(%)] |  | 5 (9.1) |  | 2 (18.2) | 0.721 |
| Bronchopulmonary dysplasia with pulmonary infection [n(%)] |  | 4 (7.3) |  | 0 | 1.000 |
| Laboratory values | | | | | |
| CRP [mg/L, ref. 0–10 mg/L], *P*_50_ (*P*_25_, *P*_75_) | 55 | 4.00 (0.80, 2.20) | 11 | 18.50 (1.60, 49.60) | 0.414 |
| PCT [ref. 0–0.05, *P*_50_ (*P*_25_, *P*_75_)] | 55 | 0.33 (0.14, 1.74) | 11 | 1.14 (0.28, 4.07) | 0.973 |
| Blood lactate [mmol/L, ref. 0.63–2.44mmo/L], *P*_50_ (*P*_25_, *P*_75_) | 55 | 1.80 (1.30, 3.10) | 11 | 1.80 (0.96, 2.70) | 0.599 |
| pH [ref. 7.35–7.45, mean±SD] | 55 | 7.38±0.07 | 11 | 7.37±0.07 | 0.782 |
| PaCO_2_ [mmHg, ref. 35–45mmHg] | | | | | |
| Before-treatment [mean±SD] | 55 | 39.19±8.57 | 11 | 43.58±10.73 | 0.142 |
| Post-treatment | | | | | |
| 2 h [*P*_50_ (*P*_25_, *P*_75_)] | 54 | 40.80 (35.53, 44.53) | 10 | 49.85 (37.90, 55.70) | 0.049 |
| 6 h [mean±SD] | 48 | 39.33±7.96 | 8 | 45.55±10.46 | 0.056 |
| 12 h [mean±SD] | 29 | 40.34±6.85 | 2 | 42.50±4.10 | 0.667 |
| PaO_2_ [mmHg, ref. 80–100mmHg] | | | | | |
| Before-treatment [mean±SD] | 55 | 83.46±25.15 | 11 | 72.75±17.33 | 0.183 |
| Post-treatment | | | | | |
| 2 h [mean±SD] | 54 | 100.80±33.30 | 10 | 74.02±18.66 | 0.017 |
| 6 h [mean±SD] | 48 | 101.61±26.25 | 8 | 77.96±28.40 | 0.023 |
| 12 h [mean±SD] | 29 | 108.79±21.17 | 2 | 69.30±4.10 | 0.015 |
| PaCO_2_/PaO_2_ ratio | | | | | |
| Before-treatment [mean±SD] | 55 | 0.51±0.18 | 11 | 0.60±0.11 | 0.094 |
| Post-treatment | | | | | |
| 2 h [*P*_50_ (*P*_25_, *P*_75_)] | 54 | 0.44 (0.33, 0.53) | 10 | 0.55 (0.51, 0.93) | 0.001 |
| 6 h [mean±SD] | 48 | 0.41±0.13 | 8 | 0.62±0.20 | ＜0.001 |
| 12 h [mean±SD] | 29 | 0.39±0.12 | 2 | 0.61±0.02 | 0.013 |
| P/F ratio [mmHg, ref. 400–500mmHg] | | | | | |
| Before-treatment[*P*_50_ (*P*_25_, *P*_75_)] | 55 | 192.75 (158.50, 258.25) | 11 | 146.34 (138.17, 168.00) | 0.031 |
| Post-treatment | | | | | |
| 2 h [*P*_50_ (*P*_25_, *P*_75_)] | 54 | 210.25 (176.60, 283.38) | 10 | 113.50 (99.61, 144.97) | ＜0.001 |
| 6 h [mean±SD] | 48 | 246.05±71.05 | 8 | 123.44±54.99 | ＜0.001 |
| 12 h [mean±SD] | 29 | 272.00±78.45 | 2 | 100.46±14.44 | 0.005 |
| S/F ratio | | | | | |
| Before-treatment[mean±SD] | 55 | 242.85±79.65 | 11 | 191.60±43.67 | 0.043 |
| Post-treatment | | | | | |
| 2 h [mean±SD] | 54 | 224.92±42.04 | 10 | 159.96±32.79 | ＜0.001 |
| 6 h [mean±SD] | 48 | 238.45±47.60 | 8 | 138.88±24.50 | ＜0.001 |
| 12 h [mean±SD] | 29 | 245.35±51.24 | 2 | 135.42±25.34 | 0.006 |
| ROX | | | | | |
| Before-treatment[*P*_50_ (*P*_25_, *P*_75_)] | 55 | 4.38 (3.26, 6.32) | 11 | 3.56 (3.13, 4.06) | 0.028 |
| Post-treatment | | | | | |
| 2 h [*P*_50_ (*P*_25_, *P*_75_)] | 54 | 5.32 (4.53, 7.27) | 10 | 2.95 (2.33, 3.72) | ＜0.001 |
| 6 h [*P*_50_ (*P*_25_, *P*_75_)] | 48 | 5.87 (4.90, 8.00) | 8 | 2.38 (1.96, 2.77) | ＜0.001 |
| 12 h [mean±SD] | 29 | 7.33±3.17 | 2 | 2.96±0.57 | 0.065 |
| Fractional change prior to treatment and up to 2 h post-treatment | | | | | |
| FC (PaCO_2_) [*P*_50_ (*P*_25_, *P*_75_)] | 54 | 0.04 (-0.09, 0.11) | 10 | 0.11 (0.02, 0.34) | 0.067 |
| FC (PaO_2_) [*P*_50_ (*P*_25_, *P*_75_)] | 54 | 0.21 (-0.02, 0.47) | 10 | -0.10 (-0.14, 0.23) | 0.081 |
| FC (PaCO_2_/PaO_2_ ratio) [*P*_50_ (*P*_25_, *P*_75_)] | 54 | -0.14 (-0.32, 0.12) | 10 | 0.15 (-0.11, 0.98) | 0.025 |
| FC (P/F ratio) [*P*_50_ (*P*_25_, *P*_75_)] | 54 | 0.11 (-0.19, 0.35) | 10 | -0.28 (-0.35, -0.06) | 0.014 |
| FC (S/F ratio) [*P*_50_ (*P*_25_, *P*_75_)] | 54 | 0.02 (-0.03, 0.08) | 10 | -0.18 (-0.40, -0.01) | 0.040 |
| FC (ROX) [*P*_50_ (*P*_25_, *P*_75_)] | 54 | 0.25 (-0.03, 0.55) | 10 | -0.10 (-0.46, 0.09) | 0.007 |

BMI, body mass index; GCS, Glasgow Coma Scale; PRISM, pediatric risk of mortality; CRP, C-reactive protein; PCT, procalcitonin; pH, pondus hydrogenii; PaCO_2_, arterial partial pressure of carbon dioxide; PaO_2_, arterial partial pressure of oxygen; PaCO_2_/PaO_2_, arterial partial pressure of carbon dioxide-to-arterial partial pressure of oxygen; P/F ratio, arterial partial oxygen pressure-to-fraction of inspired oxygen ratio; S/F ratio, percutaneous oxygen saturation-to-fraction of inspired oxygen ratio; ROX, ratio of percutaneous oxygen saturation and fraction of inspired oxygen to respiratory rate; FC, fractional change=(data at 2 h after high-flow nasal cannula oxygen therapy)-(data before high-flow nasal cannula oxygen therapy)/data before high-flow nasal cannula oxygen therapy.
